# Evaluation of Three Field-Based Methods for Quantifying Soil Carbon

**DOI:** 10.1371/journal.pone.0055560

**Published:** 2013-01-31

**Authors:** Roberto C. Izaurralde, Charles W. Rice, Lucian Wielopolski, Michael H. Ebinger, James B. Reeves, Allison M. Thomson, Ronny Harris, Barry Francis, Sudeep Mitra, Aaron G. Rappaport, Jorge D. Etchevers, Kenneth D. Sayre, Bram Govaerts, Gregory W. McCarty

**Affiliations:** 1 Joint Global Change Research Institute, Pacific Northwest National Laboratory and University of Maryland, College Park, Maryland, United States of America; 2 Kansas State University, Department of Agronomy, Manhattan, Kansas, United States of America; 3 Brookhaven National Laboratory, Department of Environmental Sciences, Upton, New York, United States of America; 4 Los Alamos National Laboratory, Los Alamos, New Mexico, United States of America; 5 EMBUL, ARS, USDA, Beltsville, Maryland, United States of America; 6 Rappaport and Associates, c/o Joint Global Change Research Institute, College Park, Maryland, United States of America; 7 Soil Fertility Laboratory, Natural Resources Institute, Colegio de Postgraduados, Carretera México-Texcoco, México; 8 CIMMYT, Km. 45, Carretera México-Veracruz, Texcoco, México, México; 9 HRSL, ARS, USDA, BARC West, Beltsville, Maryland, United States of America; Dowling College, United States of America

## Abstract

Three advanced technologies to measure soil carbon (C) density (g C m^−2^) are deployed in the field and the results compared against those obtained by the dry combustion (DC) method. The advanced methods are: a) Laser Induced Breakdown Spectroscopy (LIBS), b) Diffuse Reflectance Fourier Transform Infrared Spectroscopy (DRIFTS), and c) Inelastic Neutron Scattering (INS). The measurements and soil samples were acquired at Beltsville, MD, USA and at Centro International para el Mejoramiento del Maíz y el Trigo (CIMMYT) at El Batán, Mexico. At Beltsville, soil samples were extracted at three depth intervals (0–5, 5–15, and 15–30 cm) and processed for analysis in the field with the LIBS and DRIFTS instruments. The INS instrument determined soil C density to a depth of 30 cm via scanning and stationary measurements. Subsequently, soil core samples were analyzed in the laboratory for soil bulk density (kg m^−3^), C concentration (g kg^−1^) by DC, and results reported as soil C density (kg m^−2^). Results from each technique were derived independently and contributed to a blind test against results from the reference (DC) method. A similar procedure was employed at CIMMYT in Mexico employing but only with the LIBS and DRIFTS instruments. Following conversion to common units, we found that the LIBS, DRIFTS, and INS results can be compared directly with those obtained by the DC method. The first two methods and the standard DC require soil sampling and need soil bulk density information to convert soil C concentrations to soil C densities while the INS method does not require soil sampling. We conclude that, in comparison with the DC method, the three instruments (a) showed acceptable performances although further work is needed to improve calibration techniques and (b) demonstrated their portability and their capacity to perform under field conditions.

## Introduction

Terrestrial C sequestration through planned changes in land use and management practices has been identified as an early adoption technology to mitigate the buildup of atmospheric CO_2_ [1]–[3]. The potential sequestration is large in agricultural soils due to the large historical losses experienced by agroecosystems [1]. Many agricultural practices (e.g., diversified crop rotations, no tillage, nutrient management, and reduced irrigation practice) alone or in combination can result in soil C sequestration [1], [4]. An additional benefit of soil C sequestration is that these practices also enhance long-term soil quality and productivity [5]. For successful mitigation of atmospheric CO_2_, C sequestration practices must be implemented widely, in developing as well as developed nations, and their success monitored at different scales (e.g. farm, region, and national scales) [6], [7].

Changes in soil organic C stocks can be measured directly using soil sampling protocols and chemical analysis or estimated indirectly through the use of eddy covariance methods, stratified accounting procedures, or simulation models [2]. The standard protocol for measuring soil C changes involves soil sampling at the field and preparation for laboratory analysis. Soil C concentration is analyzed using dry combustion (DC); a method considered as the standard method due to the vast experience acquired using it and its precision [8]. The results are presented on a mass basis C per unit mass [g C kg^−1^] or per unit volume [kg C m^−3^]; alternatively they are reported as C density per unit area to a depth of 30 cm, [kg C m^−2^]) [9], [10]. Although this procedure produces excellent results, it must be done in the laboratory increasing the time, efforts and costs that restrict its routine use in agricultural C sequestration projects, particularly in developing countries. Thus, there is a need to develop portable, rapid, precise, and cost-efficient methods for measuring soil C changes in the field [10], [11].

Several technologies have been identified as potentially useful and adaptable for in-field measurement of soil C including: (a) laser-induced breakdown spectroscopy (LIBS) [12], (b) diffuse reflectance mid-infrared Fourier transform spectroscopy (DRIFTS) [13], and c) inelastic neutron scattering (INS) [14]–[16]. These three technologies—LIBS, DRIFTS, and INS—have been used extensively for elemental and chemical analysis in other applications. For example, LIBS has been used to quantify heavy metal contamination in soils [17], DRIFTS has been applied to characterize compounds and measure their concentrations in many materials [18]–[20], and INS has been employed to measure whole patient elemental composition [21]. Due to their physical characteristics, soil volumes analyzed by LIBS and DRIFTS are very small in comparison to the very large volume analyzed by INS, which does not require soil sampling.

The objective of this research is to evaluate in-field measurements of soil C densities determined by: a) LIBS, b) DRIFTS, and c) INS with laboratory determinations of C densities using the DC method.

## Materials and Methods

### Instruments

The three novel analytical methodologies for C analysis in soil, used in this work, are based on fundamentally different physical principles with vastly different characteristics and capabilities. They are LIBS, DRIFTS and INS and are described hereafter.

### Laser Induced Breakdown Spectroscopy

**Figure 1:**
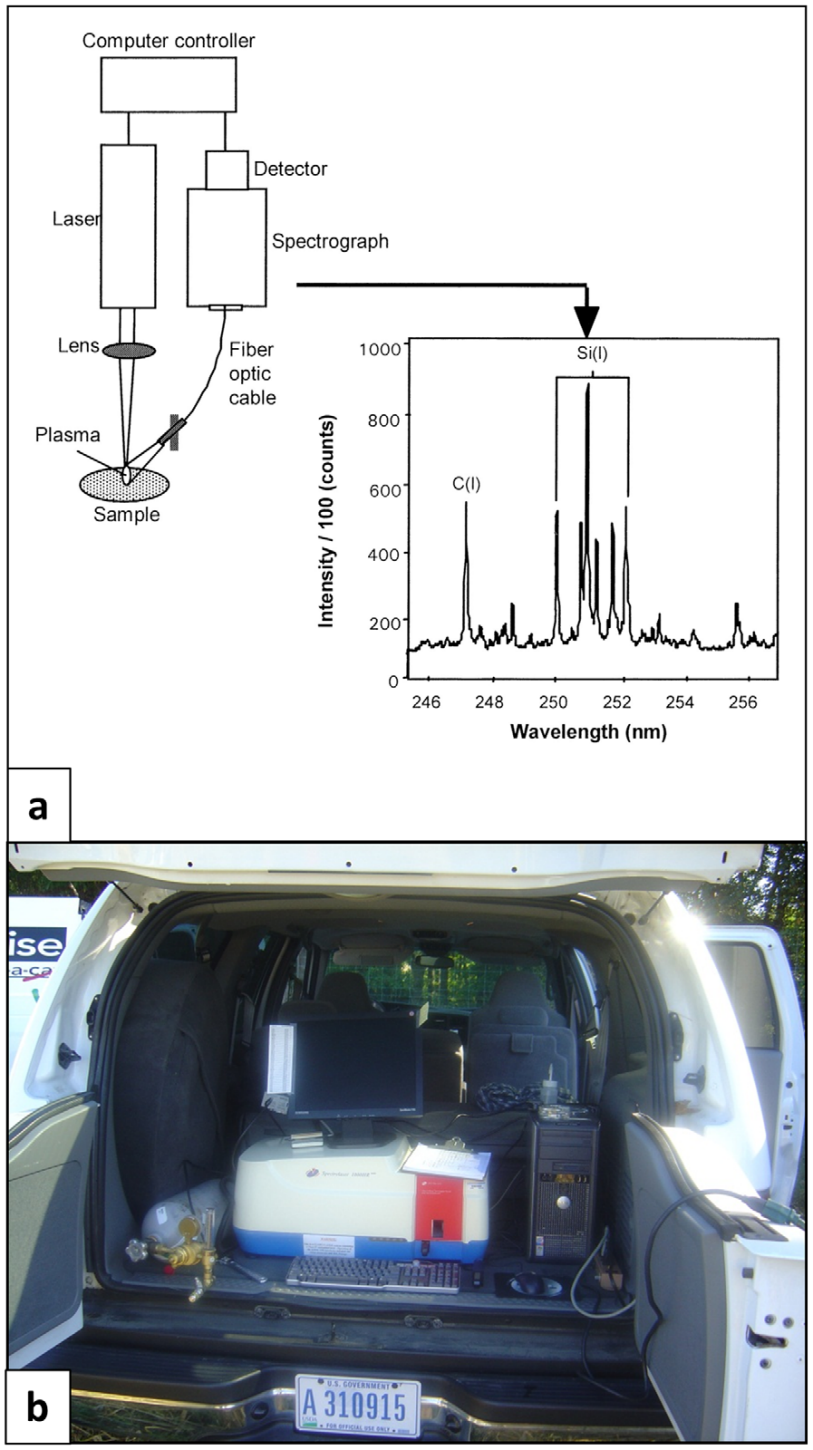
Schematic diagram and field setup of Laser Induced Breakdown Spectroscopy (LIBS) instrument: (a) schematic diagram and (b) picture of SUV-portable LIBS equipment used in this study.

The LIBS technique is based on atomic emission spectroscopy (Figure 1a) [22]–[26]. A laser pulse is focused on a (soil) sample, creating high temperatures and electric fields that break all chemical bonds in a small volume of about 10^−9^ m^3^ of the material and vaporize it into a white-hot gas of atomic ions known as microplasma [27]. The resulting emission spectrum is then analyzed using a spectrometer covering a spectral range from 190 to 1,000 nm. In order to reduce the error in the C determination it is normalized by the sum of the Al and Si intensities and taken as the standardized LIBS signal [28]. The C mass concentration was estimated from a linear fit of LIBS intensity ratio vs. C concentration on a mass basis (as determined by DC) obtained from similar soils.

Cremers et al. evaluated LIBS by measuring the total soil C of agricultural soils from Colorado and a woodland soil from New Mexico, using a subset of the Colorado samples for calibration [12]. Their tests revealed that the LIBS instrument has a detection limit of 300 mg C kg^−1^, a precision of 4–5% and accuracy of 3–14% (≈750 mg C kg^−1^), numbers some 30–400 times higher (i.e. less precise) than the same figures from DC.

The LIBS instrument has several significant operational advantages over DC. Cremers et al. found that its throughput was less than 1 minute per sample [11]. Commercially-available portable LIBS equipment was used for the tests described in this study greatly facilitating in-field measurements (Figure 1b). The LIBS method shares with the DC method the disadvantage of requiring soil core samples that are dried, mixed, sieved, and—if carbonates are present—acid-washed. The LIBS samples must be ground and pressed into pellet form, steps that are not necessary for the DC method. If maximum accuracy is not required, an un-dried whole soil core can be scanned along its length, causing the soil inside the footprint to dry, and results from different areas on the core's surface can be combined to provide statistical mixing of the sample. In this mode LIBS can provide ≈1 mm resolution (i.e., each 1 mm from top to bottom of the core).

### Diffuse Reflectance Fourier Transform Infrared Spectroscopy

**Figure 2:**
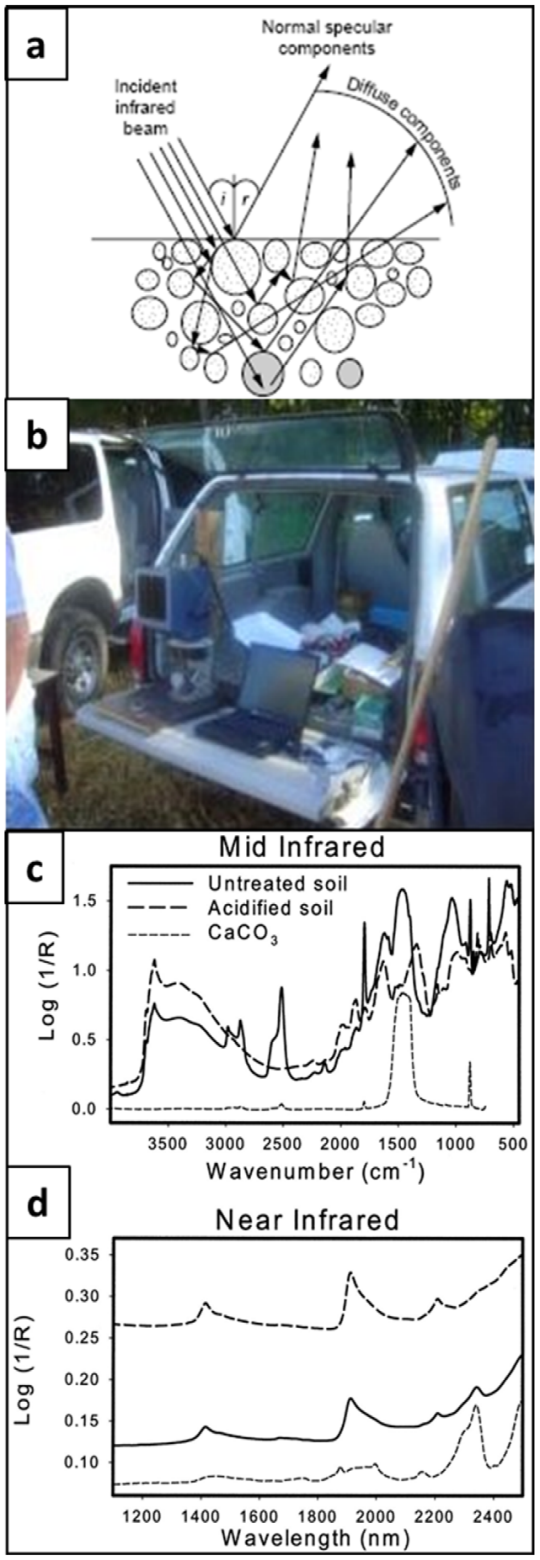
Spectra characteristics and field setup of Diffuse Reflectance Fourier Transform Infrared Spectroscopy (DRIFTS) instrument: (a) Diagram of diffuse reflection of IR light by soil sample, (b) SUV-portable mid-infrared (MIR) spectrometer used in this study, (c) typical mid-infrared diffuse reflectance spectra from soil and (d) near-infrared diffuse reflectance spectra from soil.

Unlike LIBS and INS, which probe the elemental identity of a sample's atoms, infrared (IR) spectroscopy probes the C bond identities of a sample's molecules. For soil studies, the surface of a sample is illuminated by a broadband IR source and the absorbance spectrum of the diffusely reflected component of the light is acquired by an IR spectrometer. The diffusely reflected component is light that has entered the sample and is scattered out through the same surface (Figure 2a). Like LIBS, DRIFTS and near-infrared spectroscopy (NIRS) have the operational advantages over DC of rapid throughput (1.5 to 3 minutes per sample) and portability (Figure 2b).

Many quantitative IR studies of soil – to measure component concentrations - have used the near infrared (NIR) (400–2500 nm) while qualitative investigations – to determine component chemical identities - have used the sharper absorption peaks typical of the mid- infrared (MIR) (2500–25,000 nm). The preference to use NIR over MIR is due to the weaker absorption by NIR, which would result in a longer path in the sample and hence more accurate estimates of the concentration. However, recent investigations have found that the MIR can be used for quantitative studies of low concentration components (e.g. C) in highly diverse materials such as soil [28]–[30]. Advanced data analyses techniques, such as partial least squares regression (PLSR), are essential for quantifying C content from the NIR or MIR spectra of undiluted samples. McCarty et al. tested the SOC-predictive performance of both the NIR and MIR wavelength ranges, on 237 soil samples taken from 14 locations in the U.S. Great Plains and demonstrated that MIR outperforms NIR by about a factor of two in precision [30].

Furthermore, because of its bond-sensitive nature, IR spectroscopy offers the possibility of directly distinguishing inorganic from organic C, thus eliminating the need of acid pretreatment to remove inorganic C (Figures 2c and 2d). However, McCarty et al. found that such direct estimation of organic C from MIR spectra produced root mean square errors (RMSE) 50% greater than those by DC [30]. Estimation of organic C by MIR spectra on soil samples that had undergone acid pretreatment had RMSE similar to those determined by DC. The field unit consisted of a Surface Optics Corporation model SOC-400 portable Fourier Transform spectrometer (SOC-400, Surface Optics, Corp., San Diego, CA). It is equipped with a non–Peltier cooled DTGS (deuterated triglycine sulfate) and KBr beam splitter by diffuse reflectance from 4000 to 400 cm^−1^ at 8 cm^−1^ resolution using a rotating sample cup (approximate path 2 mm in width around an 8 mm diam.) with KBr used for the background spectra. The samples spend about 45 s on the spectrometer, a time similar to that required in the lab for DRIFTS or NIRS.

### Inelastic Neutron Scattering

The INS method is based on spectroscopy of gamma rays induced by nuclear reactions of fast neutrons with nuclei of the elements present in soil. For that purpose, a portable commercial neutron generator (NG) consisting of a small (2.5 cm diameter, 10 cm long), sealed-tube accelerator produces fast, 14 MeV, neutrons by accelerating deuterium (d) impinging on a target saturated with tritium (t) resulting in a d,t fusion reaction that emits alpha and anti-parallel neutron particles [31]. The fast neutrons interacting via INS processes induce 4.43 and 6.13 MeV gamma rays in C and O atoms, respectively. Alternatively, following elastic scatterings, some of the fast neutrons slowdown and undergo capture via thermal neutron capture (TNC) reactions; inducing a 2.2 MeV gamma ray in H. These gamma rays are detected by an array of NaI detectors separated from the NG by shielding material. The entire system is mounted on a cart and non-destructively probes the soil in static and scanning modes of operation. The intensity of the measured gamma rays is proportional to the soil's C and other elements concentrations in the interrogated volume [14], [22].

**Figure 3:**
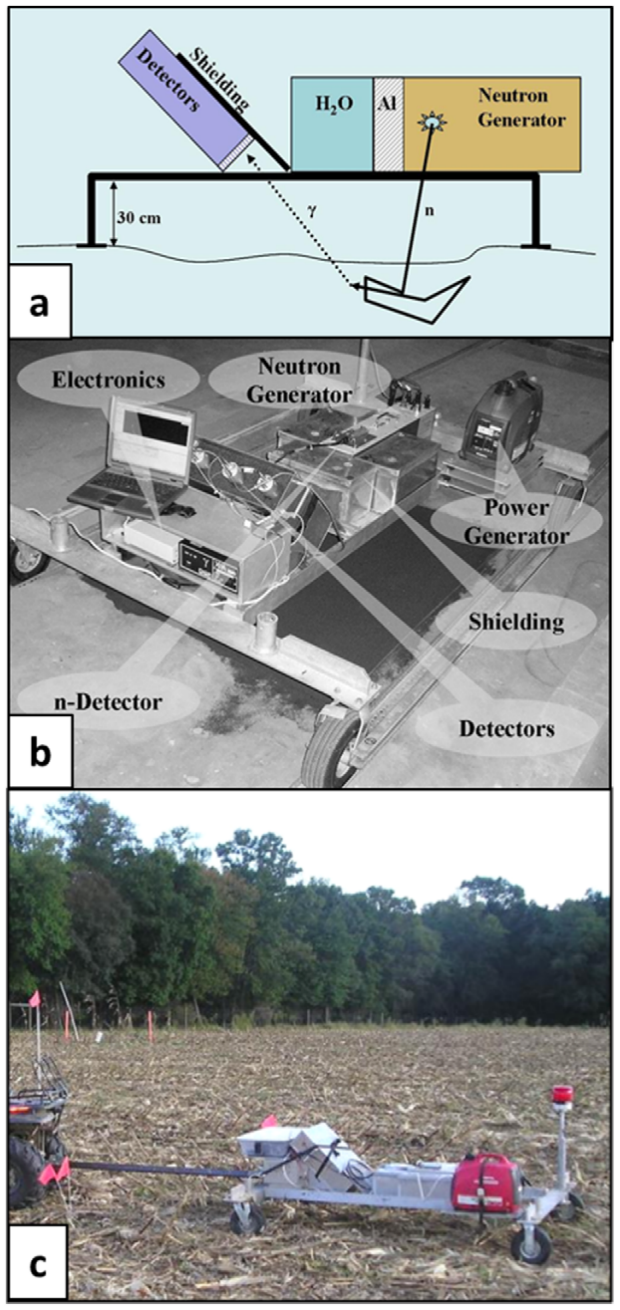
Deployment modes, schematic diagram, and field setup of Inelastic Neutron Scattering (INS) instrument: (a) The three-detector INS instrument in its various deployment modes: (a) Schematic diagram of a stationary INS instrument for soil studies, (b) the INS instrument used in this study, mounted on a cart for operation in field-scanning mode, and (c) INS instrument being towed behind a tractor during a field scan.

The high energy neutrons and gamma rays penetrate the soil quite extensively inducing detectable signal from a depth of 30 to 50 cm. The footprint of the INS system is about 1.5 m^2^ resulting in a sampled volume >0.3 m^3^. Schematics and an alpha prototype of the INS system are shown in Figures 3a and 3b; whereas Figure 3c shows the INS's scanning capability. A field scan represents a mean C value in the scanned area and the C signal from such large volumes averages any strong variations in the C depth profile.

The C intensity, the net area under the C gamma-ray peak, in measured spectra is calibrated against surface C density (g C m^−2^) of synthetic soils with known amounts of C. Calibration using synthetic soils demonstrated linearity of the INS system [14]. Alternatively in the field, the INS system is calibrated directly against chemical analysis of soil samples. However, any calibration in the field has to be concerned with comparing C content in different volumes [32]. Three advantages of the INS system include: a) it is a non-destructive method, b) it does not require sample preparation, and c) it is capable of analyzing large volumes of soil and scanning large areas [33], [34]. Further work is required to establish the sensitivity of the INS system to detect small changes in soil C content and its sensitivity to variations in C distribution with soil depth. Furthermore, the INS's ability to quantify inorganic C using the presence of Ca peak and stoichiometric information needs to be demonstrated.

### Sites and Sampling

No specific permits were required to access and sample the described field studies described below. Access to the Beltsville Experiment was coordinated by co-authors J.B. Reeves and G.W. McCarty from USDA. Access to the El Batán Experiment was coordinated by co-authors K.D. Sayre and B. Govaerts from CIMMYT. Neither location was privately-owned or protected in any way. The field studies did not involve endangered or protected species.

### Beltsville Experiment

The first side-by-side test was conducted on October 2–3, 2006 on a 25-ha USDA field (39°1′44.1″N; 76°50′41.7″W) in Beltsville, MD known as OPE3 (Optimizing Production Inputs for Economic and Environmental Enhancement) [35]. This field, contained within a first order agricultural catchment in the Maryland inner coastal plain, had been previously sampled using a 25-m grid pattern for the determination of soil fertility parameters including soil organic carbon using both near and MIR spectroscopy and then mapped by use of ordinary kriging [30]. At the time of the test, maize (*Zea mays* L.) had been recently harvested from the field, which showed a significant, but unmeasured amount of corn stover.

The experimental area was on a fine-loamy, mixed, semiactive, mesic Typic Fragiudult and the design consisted of three 30 m×30 m plots (R1, R2, and R3) where 9 sampling points were laid out on a square grid with each point placed 9-m apart from each other. At each grid point, six soil samples were extracted at three depth intervals (0–5, 5–15, and 15–30 cm) with a hand-held soil sampler (3.12 cm diam.) and composited into one sample per depth interval per grid point. The composite soil samples were taken to the side of the experimental area for further processing and measuring. In addition two extra soil samples per plot were taken for comparison with INS stationary measurements.

At the measuring station, the samples were processed and analyzed by the LIBS and DRIFTS instruments. Electric power was supplied from a nearby power source (120 V, 60 Hz). Alternatively, they can be successfully operated from a power inverter off the car battery.

After determining wet soil weight, each soil sample was manipulated in order to break the soil aggregates as well as to remove from the sample visible pieces of roots and crop residues. After each sample had been thoroughly mixed, a subsample was taken for subsequent laboratory determinations of soil water content, coarse fragment fraction, and soil C concentration by DC. For the DRIFTS analysis, soil samples were broken up and thoroughly mixed in a plastic Petri dish using a metal spatula (possible at BARC due to the sandy nature of the soils), mixed and scanned. Samples for LIBS analysis were pressed in a hydraulic press to about 20 Mg force and then placed in the LIBS sample holder. The ambient air of the LIBS instrument was replaced with Ar gas to enhance the LIBS signal, and the sample was analyzed for 10 s, which provided 100 spectra per sample. The averaged spectra were collected for further quantification of soil carbon.

The INS system was operated in stationary and scanning modes and in either case the data was acquired for one hour. For the stationary measurements, the INS instrument was placed on top of the previously sampled locations (two per each plot), subsequently an area of 900 m^2^ was scanned (Figure 3). Due to time constraints and technical issues, only Plot No. 3 was scanned. Correlation between INS readings in stationary mode and surface C density were used to develop a conversion factor for predicting soil C density of Plot No. 3.

All soil samples were analyzed for C at the Kansas State University (KSU) Laboratory by DC using a Carlo Erba C/N Analyzer (Carlo Erba Instruments, Milan, Italy) [35]. Carbon concentration results were converted to soil C density using soil bulk density values determined by the soil core method.

### El Batán Experiment

The side-by-side test in Mexico was conducted on a 17-year old crop rotation, tillage, residue study plot located at CIMMYT, at El Batán, about 40 miles east of Mexico DF and 2240 m above sea level [36], [37]. The experimental units (a total of 32) consisted of plots (22 m×7.5 m) cropped to maize (*Zea mays* L.) and wheat (*Triticum aestivum* L.), either in monoculture or in rotation, with conventional or no tillage methods, with or without crop residue removal after harvest, and with crops planted in either flat or raised beds. The plots are arranged in a randomized complete block design with two replications. All treatments planted using a flatbed system (16 treatments or 32 plots) were selected for this test.

Custom, export and import permits required by the U.S. and Mexican authorities to transfer the INS system, due to NG being a radiation producing device, precluded the participation of the INS in the Mexican tests. Thus, only the LIBS and DRIFTS techniques were tested at CIMMYT. Prior to the field test at this facility, CIMMYT researchers provided the instrument team with eight soil samples previously taken from the plots in order to facilitate the initial calibration of the LIBS and DRIFTS instruments.

A composite soil sample made of 12 subsamples per soil depth (0–5, 5–10, and 10–20 cm) was taken from each of the 32 plots. Once extracted, the soil samples were taken to an improvised processing and measuring station located about 200 m away from the plots. As in the Beltsville experiment, the composite samples were thoroughly mixed and a subsample was weighed and taken to a dry lab nearby for soil moisture determination. After separating visible pieces of crop residues and roots from the soil samples, these were set to dry, but due to the high clay content the samples dried into rock hard masses. Thus, all samples in the Mexico trials were grounded with mortar and pestle for LIBS, DRIFTS, and DC analyses. Sample preparation for LIBS analysis was essentially the same as described above. The more intense sunlight in the CIMMYT trials, however, allowed for more thorough drying of the samples before analysis.

The rest of the samples were air dried and sent to the soils laboratory at Kansas State University for DC analysis. Soil samples were finely ground to pass 100 mesh in an agate mill. Finely ground soil samples were oven dried to 105°C for 24 h and stored. Just before analysis, the samples were oven dried again for 2–3 h, removed from the oven and placed in a desiccator prior to analysis. The amount of total C was determined by DC (900°C) in an automatic C analyzer Shimadzu TOC 5000-A (Shimadzu Scientific, Kyoto, Japan).

### Statistical Analysis

Statistical analysis consisted of regression and ANOVA analysis using SAS software (SAS Institute Inc., Cary, NC). Data for Beltsville experiment were analyzed using a Completely Randomized Design. Data for the Mexican experiment were analyzed following a Randomized Complete Block Design.

### Ethics Statement

No specific permits were required to access, perform the studies, and sample the field experiments at Beltsville (Maryland) in the USA and at CIMMYT, El Batán in Mexico. At Beltsville, the technical group was allowed access to the OPE3 field by USDA – ARS scientist and team member J.B. Reeves III. At CIMMYT, the technical group was allowed access to the long-term trial by CIMMYT scientist and team member K.D. Sayre. Soil sampling conducted at both sites followed standard protocols. Soil samples taken at CIMMYT were imported to the USA by KSU professor, permit-holder, and team member C.W. Rice for analysis. Transport of equipment across interstate and international borders followed all state, federal, and international regulations.

## Results

### Beltsville Experiment

**Figure 4:**
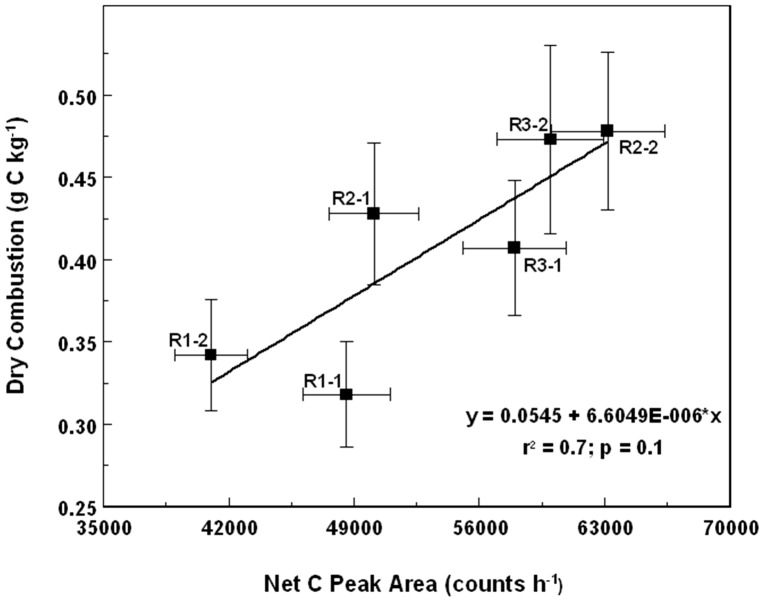
Correlation between INS signal and soil C density as measured by dry combustion to a depth of 30 cm in Beltsville, MD.

The mean soil C density of Plot No. 3 as determined by DC was 4.07±0.55 g C m^−2^ (Table 1). A relatively low coefficient of variation of 14% suggests a rather uniform distribution of C within the estimated plot volume (270 m^3^). The soil C density estimate by LIBS was about 20% lower than that by DC (p<0.01) while the DRIFTS estimate was not different from that determined by DC (p<0.83). In the case of INS, two values are provided (Table 1): one using a “universal” calibration and the other using a “local” calibration. Use of a “universal” calibration produced a mean estimate of soil C density about 37% lower than that obtained by the DC method. Since INS provides a single value for the entire field with a counting statistics error of about 1.5%, it requires more replications for comparing against other methods using conventional statistics. Additional “stationary” measurements at six other sites in the experimental area allowed for the development of a regression line between the DC and INS methods (Figure 4). When using the “local” calibration, there was almost a perfect agreement between the DC and INS methods.

**Table 1:** Soil-C density statistics to a depth of 30 cm of Plot No. 3 at the OPE3 field in Beltsville, MD as determined by dry combustion and three soil-C technologies.

				INS	
	DC	DRIFTS	LIBS	Universal calibration	Local calibration
	------------------------------------------- kg C m^−2^ ------------------------------------------------------------------------------------------------------------------------------------				
Mean	4.07	4.32	3.27	2.57	4.06
Std. Dev.	0.55	0.61	0.81	0.61	0.23
	----------------------------------------------------------------------------------------------------------------------------------------------------------------------------------------------				
Dev. (%) from DC	—	6.14	−19.7	−36.9	−0.3
No. samples	9	9	9	Soil volume scanning	

Table 2 provides a more detailed statistical comparison of the soil C density values determined by DC, LIBS and DRIFTS for the three depths and total soil depths on Plot No. 3. Again, for the three depths, the soil C density derived from DRIFTS measurements was not significantly different from those by DC. In this case, the enhanced calibration dataset used by the DRIFTS team contributed to the close results.

**Table 2:** Analysis of Variance of Dry Combustion, LIBS, and DRIFTS soil C density means of Plot No. 3 at the OPE3 field in Beltsville, Maryland.

	Depth interval							
	0–5		5–15		15–30		0–30	
	Mean Square	Pr>F	Mean Square	Pr>F	Mean Square	Pr>F	Mean Square	Pr>F
Treatment	0.1289	0.001	0.1825	0.051	0.7886	0.093	2.6965	0.007
Error	0.0142		0.0542		0.3005		0.4455	
R^2^	0.430		0.219		0.179		0.335	
	Soil Carbon Means (kg C m^−2^)							
Dry Comb.	0.86	a†	1.76	a	1.45	ab	4.07	a
LIBS	0.68	b	1.50	b	1.09	b	3.27	b
DRIFTS	0.91	a	1.73	a	1.67	a	4.32	a

† Means followed within depth interval followed by the same letter are not significantly different at the 0.05 level of probability.

### El Batán Experiment

The DC measurements were repeated campus of the Colegio de Postgraduados at Montecillo, Mexico, affording a measurement of the operational accuracy (repeatability *between* instruments) of this “gold standard” technique for measuring soil carbon.

Table 3 summarizes results of the Analysis of Variance conducted on soil C density calculated to a depth of 20 cm by three methods: DC, LIBS, and DRIFTS. Here we used a Randomized Complete Block design with three main factors: tillage, rotation, and residue with two replications. Overall, R^2^ was largest for the DC dataset, followed by LIBS, and DRIFTS. The DC method detected significant differences due to tillage and residue effects while the LIBS and DRIFTS methods detected significant differences only due to tillage effects. Further, a one-way ANOVA analysis (bottom half of Table 3) reveals the DC method with higher sensitivity that the other two methods to detect significant differences among means.

**Table 3:** Summary of ANOVA showing mean square values for main effects, main effect means, and overall means for the 16 treatments with two replications analyzed for soil C density (kg C m^−2^) at El Batán, Mexico in 2007.

Source	Mean Square	Pr.>F	Mean Square	Pr.>F	Mean Square	Pr.>F
	Dry Comb.		LIBS		DRIFTS	
Tillage	2.3992	0.002	5.6890	0.009	3.0928	0.0003
Rotation	0.0143	0.752	1.2217	0.202	0.0151	0.772
Residue	1.7424	0.002	1.2332	0.200	0.0150	0.772
Error	0.1392		0.7085		0.1752	
R^2^	0.649		0.590		0.537	
	Tillage					
CT	2.72	b	2.81	b	2.91	b
ZT	3.26	a	3.66	a	3.53	a
	Rotation					
Monoculture	3.01	a	3.43	a	3.24	a
Rotation	2.97	a	3.04	a	3.20	a
	Residue					
Retained	3.22	a	3.43	a	3.24	a
Removed	2.76	b	3.04	a	3.20	a

Treatment variables				Soil Carbon Determination Method					
Tillage	Rotation	Crop	Residue	Dry Comb.		LIBS		MIRS	
CT	Monoculture	Maize	Retained	3.39	abc†	3.83	ab	3.53	abc
		Maize	Removed	2.35	e	2.93	abcd	2.82	c
		Wheat	Retained	2.64	de	2.52	bcd	2.83	c
		Wheat	Removed	2.57	de	3.29	abcd	2.79	c
	Rotation	Maize	Retained	2.97	abcde	3.21	abcd	2.93	bc
		Maize	Removed	2.56	e	2.58	bcd	2.82	c
		Wheat	Retained	2.79	cde	2.27	cd	2.81	c
		Wheat	Removed	2.46	e	1.89	d	2.74	c
ZT	Monoculture	Maize	Retained	3.52	ab	3.91	ab	3.46	abc
		Maize	Removed	2.91	bcde	2.63	bcd	3.37	abc
		Wheat	Retained	3.45	abc	4.36	a	3.48	abc
		Wheat	Removed	3.26	abcd	3.99	ab	3.65	ab
	Rotation	Maize	Retained	3.64	a	3.97	ab	3.63	ab
		Maize	Removed	3.01	abcde	3.54	abc	3.43	abc
		Wheat	Retained	3.39	abc	3.40	abc	3.26	abc
		Wheat	Removed	2.93	bcde	3.47	abc	3.97	a

† Means within a given method followed by the same letter are not significantly different at the 0.005 level of probability.

**Figure 5:**
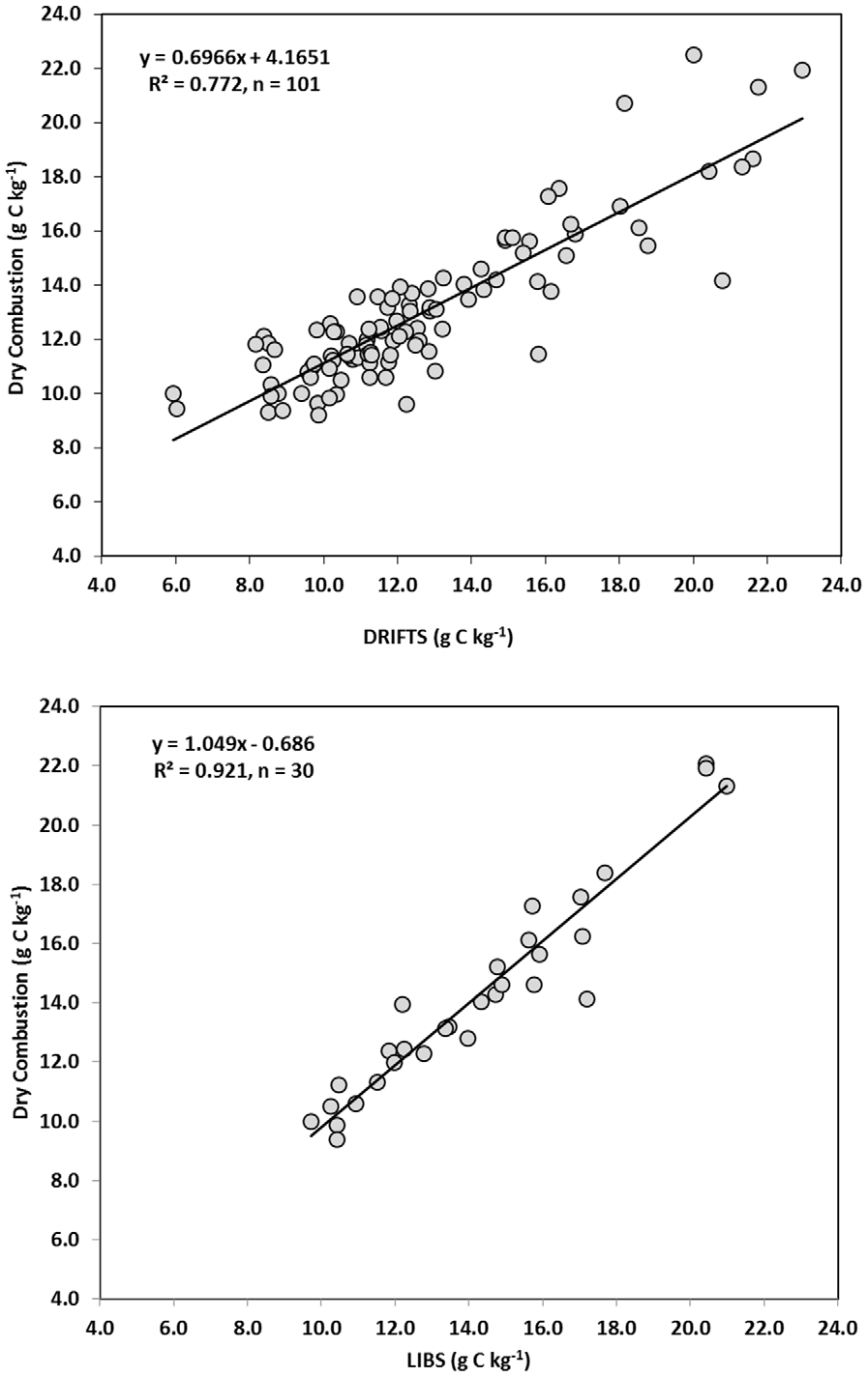
Comparison of calibration lines for (a) DRIFTS and (b) LIBS made by including 10% of the data in the calibration sets (see text).

Subsequently, the LIBS and DRIFTS teams were provided with C concentrations for 11 (10%) of the samples to augment their respective calibration curves. The DRIFTS team used these samples from the dataset, eight samples from archived soil samples taken from the experimental site, and all of the data from OPE3 trial. Using these extra points in the calibration curve the R^2^ for the DRIFTS instrument improved significantly to 0.772 (Figure 5). Using the same 11 samples to construct their new calibration curve, and using PLS methods, the LIBS team improved their instrument's R^2^ to 0.919 although they were only able to use 30 of the 101 samples due to software limitations (Figure 5). These results demonstrate that there is considerable opportunity to improve the predictability of both instruments by using DC results from a small number of local samples.

**Figure 6:**
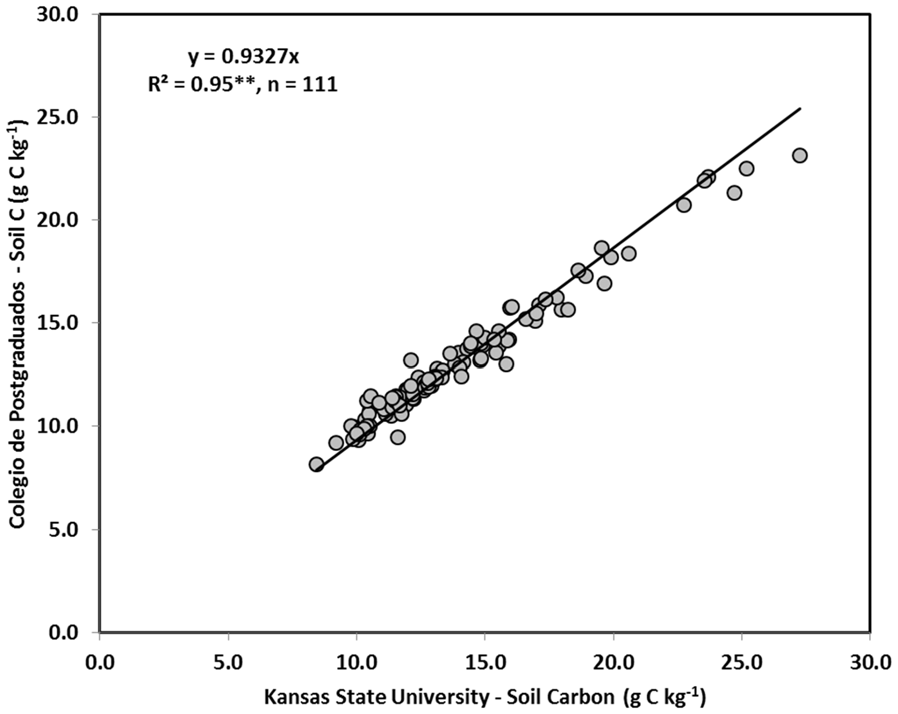
Comparison of dry combustion results from the two different instruments used.

The DC results from the KSU and Colegio de Postgraduados correlated rather well. Figure 6 shows linear fits of the DC values obtained at Colegio de Postgraduados to the DC values obtained at KSU. The R^2^s obtained were 0.97 and 0.95, depending on whether or not a non-zero intercept was allowed between the results of the two instruments. The median value for the deviation between the two measurements, expressed as a percentage of the average of the measurements, was 5.4%, reflecting that the two medians were 3 standard deviations away from each other. These discrepancies are in accord with previous findings [6].

## Discussion

Based on the results of the blind comparison in Beltsville, the DRIFTS instrument produced the closest estimates of soil C density for Plot No. 3 but required the largest amount of ancillary information to arrive at these results. In the case of the LIBS instrument, the estimates of soil C density could be improved by including more data points into the universal calibration curve. With regards to the INS instrument; since only single measurements yields the value for the entire field it needs to be reproduced to justify the complete agreement with the results by DC. It was pointed out that the INS instrument is calibrated directly in terms of soil C density, Figure 4, using large volumes without the need of knowing bulk density. The scarcity of data, only six points with two from each plot used for creating Figure 4, resulted in r^2^ value of 0.7, however, in two other fields studies in mixed soils the regressions coefficients were higher than 0.9 [15], [32]. Furthermore, since INS is using penetrating radiation that is exponentially attenuated it does not have a precisely defined depth of sampling. Instead, we define it as the depth from which 90% of the signal is detected. Based on Monte Carlo calculations, this effective depth is about 30 cm while for 99% it is about 50 cm. Since small signal arrives from deeper layers, variation in the depth should not play a major role in the total count [33].

The original plan was to complete the soil sampling, soil analysis in the field with the LIBS and DRIFTS instruments, as well as stationary and scanning measurements in the three plots within a period of two days (October 2–3, 2006). The activities performed during this period included: plot and sampling site demarcation, instrument setup, weighing station setup, soil sampling, preparation of samples for analysis in the field, sample analysis with LIBS and DRIFTS, stationary and scanning measurements with INS, and soil sample preparation for submission to laboratory for DC analysis. A total of 10 researchers and technicians were involved in these operations. The LIBS and DRIFTS teams were able to complete the analysis of 81 samples from the three plots and 26 samples from the stationary sites. As noted before, technical difficulties (a loose wire in the neutron generator) delayed the INS field measurements and allowed for the completion of six stationary measurements, two per plot, and two scans of plot No. 3 during the 2-day experimental period. Consequently, comparisons of the three instruments and DC were available only for one plot, Plot No. 3.

After completion of the DC analysis, some of the results were made available to the LIBS and DRIFTS teams. The DRIFTS team used results from Plot No. 1 and 2 to independently predict the values of Plot No. 3. The LIBS team did not use any of the values from Plot No. 1 and 2 to improve their “universal” calibration curve. Finally, the INS team used counts from six stationary INS measurements, two from each plot, to develop a calibration curve (soil C density (g C cm^−2^ = 54,714×INS_count – 11,026) to predict the average soil C density of Plot No. 3 to a depth of 30 cm.

In summary, this study compared the side-by-side performance of three advanced technologies to measure soil C under field conditions against standard soil carbon analysis by DC. The LIBS and DRIFTS methods and the standard DC require soil sampling and need soil bulk density information to convert soil C concentrations to soil C densities. The INS method requires some soil sampling for establishing correlations between INS and DC but no further sampling is necessary once these correlations are established. The comparative results obtained indicate an acceptable performance of the three instruments but they also show the need for improvement in terms of calibration.

In terms of transportability, the INS system is a radiation generating device and thus has to follow all transportation regulations, which, at the moment, impede international shipping. The LIBS instrument, as tested in our experiments, requires Ar gas, a press and a power source. Finally, the DRIFTS instrument was portable and can be run off a car battery or an inverter with the vehicle running. No other equipment is required other than a mortar and pestle. There are also portable MIR and NIR units available that can run off backpacks or even internal batteries.

The three instruments demonstrated their portability and their capacity to perform under field conditions. Import/export issues to developing countries (i.e., regulations, permits, and licenses) should be carefully examined in order to facilitate the smooth transport of these instruments across international borders.
